# Diffuse Reflectance Spectroscopy for Caries Detection and Discrimination of Restorative Materials: An In Vitro Diagnostic Accuracy Study

**DOI:** 10.1159/cre/abuag010

**Published:** 2026-09-10

**Authors:** Jindřich Charvát, Aleš Procházka, Lucie Himmlová, Tomáš Kučera, Lenka Vavřičková

**Affiliations:** a Institute of Dental Medicine, First Faculty of Medicine, Charles University and General University Hospital in Prague, Prague, Czech Republic; b Department of Mathematics, Informatics and Cybernetics, University of Chemistry and Technology in Prague, Prague, Czech Republic; c Czech Institute of Informatics, Robotics and Cybernetics, Czech Technical University in Prague, Prague, Czech Republic; d Institute of Histology and Embryology, First Faculty of Medicine, Charles University, Prague, Czech Republic; e Department of Dentistry, Faculty of Medicine in Hradec Králové, Charles University, Hradec Králové, Czech Republic; f Department of Dentistry, University Hospital Hradec Králové, Hradec Králové, Czech Republic

**Keywords:** Diffuse reflectance spectroscopy, Dental caries, ICDAS, Diagnostic accuracy, Restorative materials

## Abstract

**Introduction:**

Diffuse reflectance spectroscopy (DRS) is a proposed optical adjunct for caries detection, but its diagnostic accuracy against histology has not been validated alongside its ability to discriminate restorative materials. We aimed to validate DRS in vitro for both tasks.

**Methods:**

This in vitro study comprised two arms. In part 1, 200 occlusal regions of interest from 100 extracted teeth were assessed by DRS (350–1,730 nm) and by two independent examiners using the International Caries Detection and Assessment System (ICDAS), with histological scoring as the reference standard. In part 2, four freshly prepared restorative materials (45 sites each: a self-adhesive resin cement, two resin composites, a resin-modified glass-ionomer) were compared with 37 sound-enamel sites from a separate cohort (one site per tooth). Spectra were classified by partial least squares discriminant analysis (PLS-DA) with specimen-grouped 10-fold cross-validation (sites from a specimen kept in one fold, preventing leakage).

**Results:**

DRS reached 80.5% exact agreement with histology (quadratically weighted κ = 0.916, 95% CI: 0.890–0.939) versus 54.0–57.5% for the visual examiners. At the any-caries threshold (histological score ≥D1), DRS achieved sensitivity 90.8% (95% CI: 84.3–94.8) and specificity 95.0% (87.8–98.0), with four false-positive classifications of sound surfaces against 25 and 23 for ICDAS examiners. The DRS AUC (0.946) exceeded both examiners (0.908 and 0.914), reaching significance against one (DeLong test, *p* = 0.027). Sensitivity for dentin-level caries was low (37.5%). PLS-DA discriminated the five classes (four materials plus sound enamel) with overall accuracy 99.5% (95% CI: 97.4–99.9).

**Conclusion:**

In vitro, DRS showed markedly higher specificity than visual ICDAS scoring for occlusal caries and discriminated four freshly prepared restorative materials from sound enamel. Its low sensitivity for dentin-level lesions and the controlled laboratory conditions warrant cautious interpretation pending in vivo validation.

## Introduction

Dental caries remains the most prevalent noncommunicable disease worldwide [1]. Contemporary cariology has shifted from operative (“drill-and-fill”) management toward minimally invasive, risk-based care, in which non-cavitated lesions are treated as potentially reversible when detected early [2]. Yet, accuracy cuts both ways – new diagnostic aids are readily used as a license to intervene, and even remineralization-oriented management can overtreat, so high specificity and positive predictive value (PPV) matter as much as sensitivity for any caries adjunct [3].

Visual detection is anchored by the International Caries Detection and Assessment System (ICDAS), a seven-point ordinal scale validated against histology and embedded in international management frameworks [4]. Its pooled accuracy has been quantified repeatedly. The meta-analysis of Gimenez et al. [5] reported specificities mostly above 0.90 but widely varying sensitivity. The systematic review of Ekstrand et al. [6] confirmed good reproducibility for detection yet weaker performance for lesion activity. The 2021 Cochrane assessments reaffirmed reasonable but heterogeneous accuracy with wide confidence intervals [7–9]. On occlusal surfaces, visual scoring remains examiner dependent and hinges on meticulous cleaning and drying, leaving diagnostic uncertainty for early non-cavitated lesions – the window where noninvasive management matters most.

Objective adjuncts illustrate a recurring trade-off. Fluorescence-based devices such as DIAGNOdent and quantitative light-induced fluorescence add quantitative signal but stay susceptible to stain, plaque, and restorative confounders [10–12]. Near-infrared transillumination and optical coherence tomography improve visualization yet give only heterogeneous accuracy or stay research-grade owing to cost and complexity [7, 13]. Each is bound by its underlying physical principle, leaving room for a chairside-compatible method built on a different one.

Diffuse reflectance spectroscopy (DRS) rests on a different principle. Rather than emitted fluorescence, it records broadband visible and near-infrared (VIS-NIR) light that has entered the tissue, undergone absorption and scattering, and re-emerged, giving a spectral fingerprint of the mineralized hard tissue [14]. Because this signal reflects bulk mineral optics rather than surface porphyrins or plaque, DRS is less sensitive to the confounders limiting fluorescence methods. We previously showed that broadband VIS-NIR DRS with machine-learning classification exceeded ICDAS and DIAGNOdent in specificity and accuracy for enamel-confined lesions [15]. Optical differences between dental tissues and restorative materials are also known [16, 17], but no systematic DRS evaluation across clinically relevant material classes exists. The present in vitro study therefore aimed to (i) determine the accuracy of VIS-NIR DRS for occlusal caries detection in extracted permanent teeth, with ICDAS as comparator and histology as reference standard and (ii) test whether DRS separates four freshly prepared restorative materials from sound enamel. We hypothesized that DRS would be at least comparable to ICDAS against histology, and that the four materials and sound enamel would be classified well above the approximately 20% chance level expected for five near-balanced classes.

## Methods

### Study Design and Reporting

This in vitro study had two arms with independent specimens and a shared spectroscopic system. Part 1 assessed the diagnostic accuracy of DRS for occlusal caries detection on extracted teeth, using histology as the reference standard and ICDAS as the clinical comparator; part 2 assessed DRS discrimination of four direct restorative materials from sound dental hard tissue. Data were collected from January 2019 to January 2025. Part 1 is reported in accordance with the STARD 2015 guidelines [18, 19]; the completed checklist is provided as online supplementary material (for all online suppl. material, see https://doi.org/10.1159/cre/abuag010).

The use of extracted teeth was approved by the General University Hospital in Prague Ethics Committee under protocols No. 613/18 S-IV (part 1; approved April 19, 2018) and No. 1/23 GIP (part 2; approved February 24, 2023); donors had provided written informed consent for research use of the teeth. The study followed the Declaration of Helsinki, and no identifying donor information was retained.

Teeth were collected at the Institute of Dental Medicine (First Faculty of Medicine, Charles University and General University Hospital in Prague) and a collaborating practice in Statenice, Czech Republic. DRS, ICDAS, and histology were performed at the same Institute and the Institute of Histology and Embryology; data were processed at the University of Chemistry and Technology, Prague.

### Specimens

#### Tooth Specimens for Caries Detection (Part 1)

One hundred extracted permanent posterior teeth (premolars and molars) were collected sequentially from adult donors undergoing extraction for periodontal, orthodontic, or surgical reasons. Inclusion criteria were: (i) intact occlusal surface presenting as sound enamel or non-cavitated caries not exceeding ICDAS code 3, focusing the cohort on the early-detection window and (ii) absence of occlusal restorations. Exclusion criteria were crown fractures, prior endodontic treatment, restorations, advanced cavitated lesions, and developmental enamel defects (hypomineralization, fluorosis).

After extraction, teeth were stored in 0.5% chloramine-T solution for 1 week, then in weekly-renewed distilled water until measurement (≤6 months) [15]. Before assessment, each tooth was cleaned with a rotary brush and non-fluoridated paste (Depural Neo; SpofaDental, Jičín, Czech Republic) and rinsed. Chloramine-T (0.5%) is a standard storage-disinfection medium. Because the part 1 and part 2 tooth specimens underwent identical storage and cleaning, any storage-related effect on enamel optics would be consistent across the two tooth cohorts. The restorative-material specimens, however, were stored under different conditions, so a contribution of storage to the tooth-material spectral differences cannot be fully excluded.

Two pit-and-fissure regions of interest (ROIs) were predefined per tooth (200 total) by the principal investigator (J.C.), each bisected by the planned plane of histological sectioning to ensure ROI-to-section correspondence. Each ROI was photographed (Nikon D5100, 90-mm macro lens; Nikon, Tokyo, Japan) for spatial co-registration across ICDAS, DRS, and histology.

#### Tooth Specimens for Material Comparison (Part 2)

An independent cohort of 40 extracted, caries-free permanent teeth served as a sound-tissue reference (no carious lesions, restorations, cracks, or developmental defects on the buccal surface; storage and cleaning as in part 1). After quality control (QC), 37 teeth were retained; three teeth measured in a single session on December 30, 2019 were excluded because none of their ten spectra met the QC thresholds, indicating a session-specific calibration issue. A single site was defined on the mid-buccal surface – a flat enamel area geometrically comparable to the planar material specimens, minimizing geometric bias when tooth and material spectra are compared, and deliberately differing from the pit-and-fissure ROIs of part 1.

#### Restorative Material Specimens (Part 2)

Four direct restorative materials were investigated (Table 1): a self-adhesive resin cement (RelyX Unicem; 3M, St. Paul, MN, USA), a nano-hybrid composite (G-aenial Anterior, Shade A2; GC Corporation, Tokyo, Japan), a light-cured resin-modified glass-ionomer cement (Riva LC, Shade A2; SDI Limited, Bayswater, Australia), and an enamel-shade composite (Enamel Plus HRi Bio Function, shade BF2; Micerium S.p.A., Avegno, Italy). Nine cuboidal specimens (10 × 10 × 10 mm) per material were prepared in custom polytetrafluoroethylene molds. Light-cured materials were polymerized in 2-mm increments (VALO LED unit; Ultradent Products, Inc., South Jordan, UT, USA; ≈1,400 mW·cm^−2^, 20 s per increment); the self-adhesive cement was set per manufacturer’s instructions. Specimens were finished against a glass slide and stored dry for 10 days before measurement, allowing resin post-cure conversion and glass-ionomer maturation to a stable end state.

**Table 1.** Restorative materials investigated in part 2

Material category	Trade name	Manufacturer	Shade
Self-adhesive resin cement	RelyX Unicem	3M, St. Paul, MN, USA	–
Nano-hybrid composite (anterior)	G-aenial Anterior	GC Corporation, Tokyo, Japan	A2
Resin-modified glass-ionomer (light-cured)	Riva LC	SDI Limited, Bayswater, Australia	A2
Enamel-shade esthetic composite	Enamel Plus HRi Bio Function	Micerium S.p.A., Avegno, Italy	BF2

All materials were measured in their freshly polymerized, unaged state. Nine cuboidal specimens (10 × 10 × 10 mm) were prepared per material in custom polytetrafluoroethylene molds. Light-cured materials were polymerized in 2-mm increments using a VALO LED curing unit (Ultradent Products Inc., South Jordan, UT, USA) in high-power mode (≈1,400 mW·cm^−2^, 20 s per increment). Five spatially separated measurement sites were defined on each specimen, yielding 45 sites per material.

### ICDAS Clinical Examination

Visual inspection of all 200 ROIs was performed by two independent dentist examiners using ICDAS [20]. Examiner 1 (J.C., 9 years postgraduation) and Examiner 2 (L.V., 20 years postgraduation, Assistant Professor with restorative-dentistry expertise) were blinded to all other modalities (DRS, histology) and to each other’s scores. Both examiners completed the ICDAS Foundation e-learning module before the study (www.icdas.org); inter-examiner agreement was quantified by quadratically weighted Cohen’s kappa (see Statistical Analysis).

Examinations followed routine clinical conditions: operatory light, no magnification, three-way air drying for 5 s, no probe, in line with current recommendations [6]. Each ROI was scored on the seven-point ICDAS scale (0–6) [21]: 0 = sound, 1–2 = non-cavitated enamel changes, 3 = localized enamel breakdown, 4–6 = cavitated lesions. Inclusion criteria precluded scores ≥4, so the analytical scale was effectively 0–3.

The photographic ROI reference ensured both examiners scored the same surface. Each worked independently; no consensus was generated, and both scores were retained for separate analysis against histology [15].

### Diffuse Reflectance Spectroscopy

#### Instrumentation and Calibration

Spectral measurements used a custom dual-spectrometer DRS console (CONSOLE10) developed by Philips Research (Eindhoven, The Netherlands) and previously validated for caries detection [15, 22]. A halogen broadband source feeds two synchronous spectrometers (Horiba S330-2 silicon, visible to 985 nm; Horiba S318-2 InGaAs, 845–1,730 nm; Horiba, Kyoto, Japan), giving a composite working range of 350–1,730 nm [15, 22]. The handheld probe contains two parallel optical fibers (0.85 mm center-to-center) for illumination and collection of back-scattered light, giving a probing depth suitable for enamel and upper-dentin demineralization [15] (Fig. 1).

**Fig. 1.**
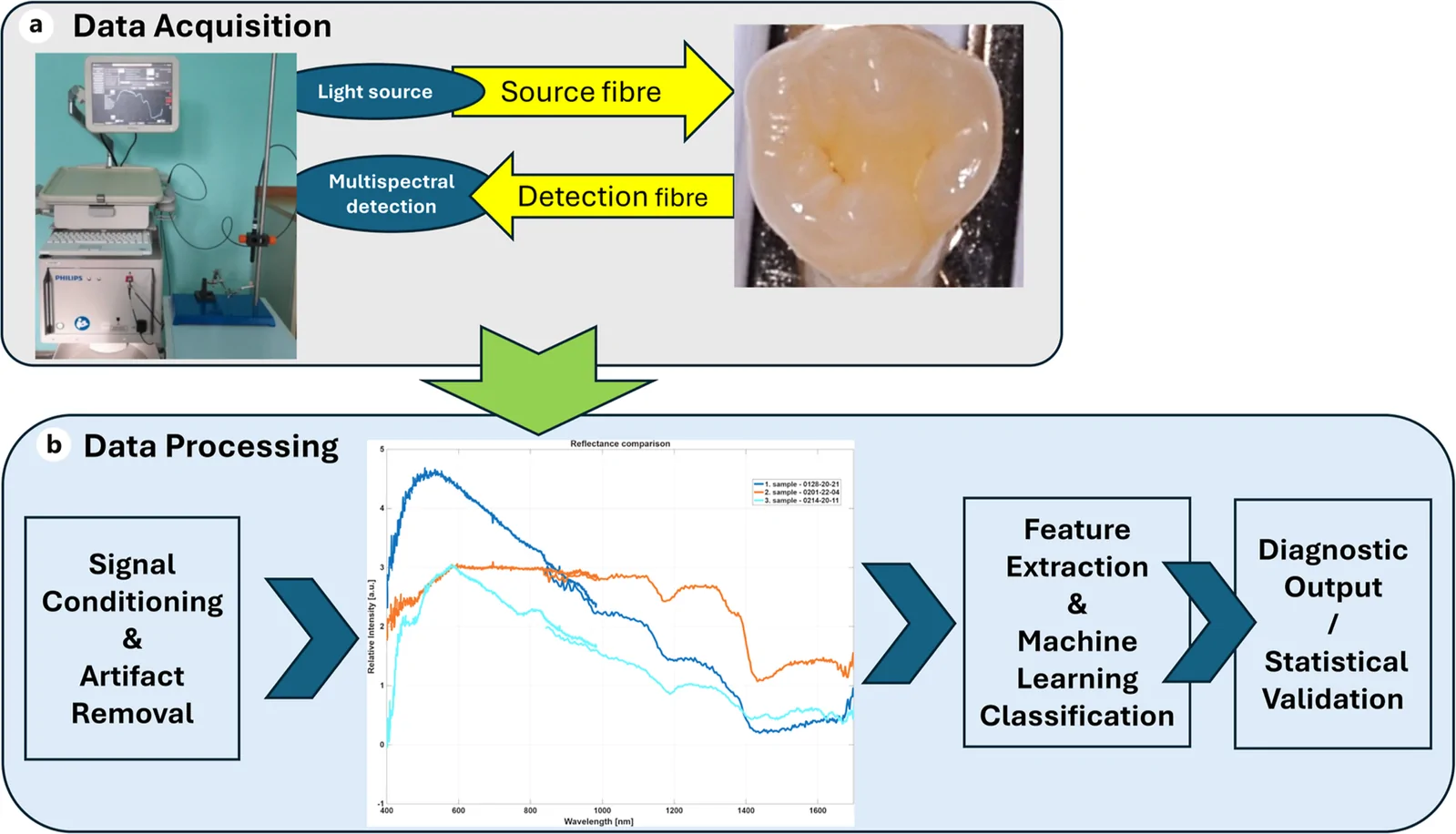
Overview of the DRS measurement and analysis workflow. **a** Data acquisition: the CONSOLE10 dual-spectrometer instrument (Philips Research, Eindhoven, The Netherlands; left inset) delivers broadband halogen illumination through a source fiber, while diffusely reflected light returns through a parallel detection fiber placed in contact with the occlusal surface (right inset; yellow arrows indicate the optical path). **b** Data processing: raw site spectra undergo calibration, QC, smoothing, SNV normalization and averaging (online suppl. Fig. S2), then feature extraction and supervised classification. DRS, diffuse reflectance spectroscopy; SNV, standard normal variate.

A four-step calibration chain – white-light reference against a 99% Spectralon standard (Edmund Optics Inc., Barrington, NJ, USA), automatic dark-current subtraction, lamp-on/lamp-off stray-light correction, and a probe-specific correction vector averaged from 50 reference scatterer spectra – was applied to every spectrum to yield calibrated reflectance R(λ), as described previously [15, 22] (full formula in online suppl. Note S1). System repeatability was verified against a fixed reference tooth at session start and end and after any change in room or illumination conditions.

#### Acquisition Parameters and Environmental Conditions

Integration times were 500 ms (VIS)/1,000 ms (NIR), one acquisition per spectrum, under manufacturer software control with temperature logging. All acquisitions ran at 20–23°C and 30–60% relative humidity to maintain stable lamp output and detector response. Tooth specimens were measured under standard laboratory illumination; material specimens in a darkened room to minimize stray light.

#### Tooth and Material Measurement Procedure

All tooth DRS measurements were performed by J.C. (blinded to ICDAS scores). Each tooth was held in a custom holder and the probe placed in gentle perpendicular contact with the predefined ROI (occlusal pit/fissure for part 1; mid-buccal flat enamel for part 2); inclination was controlled visually, as it affects reflectance [22]. Each ROI was air-dried for 5 s and measured ten consecutive times, with the probe tip wiped with isopropanol between specimens. Material specimens followed the same procedure in a mechanical holder maintaining constant probe distance and contact; five spatially separated sites were measured per cube (45 per material, 180 total), with ten spectra per site.

#### Spectral Dataset

The complete dataset comprised 2,000 spectra from 200 ROIs (part 1) and 2,200 spectra from 220 prospective sites (part 2; 180 material and 37 tooth-reference sites retained after QC); per-site mean spectra were computed during preprocessing.

### Histological Reference Standard

After ICDAS and DRS, part 1 teeth were processed for histology within 2 weeks, following our previous protocol [15]. The radicular portion was embedded in acrylic resin (Duracryl Plus; SpofaDental, Jičín, Czech Republic) and sectioned through the predefined ROIs with a water-cooled low-speed saw (IsoMet; Buehler, Esslingen, Germany), yielding two serial sections per tooth (150–200 μm). The sectioning plane was preplanned during ROI selection so each ROI was bisected by a section.

Sections were ground and polished (EcoMet 30; Buehler, Esslingen, Germany; 800-grit SiC) to optically flat specimens and examined under a Leica DMLB transmitted-light microscope at 10× (Leica Microsystems, Wetzlar, Germany). Histological scoring was performed by a single experienced histologist (T.K.), blinded to ICDAS and DRS results, using an established histological caries-grading scale [23]: 0 = sound or narrow surface opacity; 1 = outer-half enamel demineralization; 2 = inner-half enamel; 3 = outer-half dentin; 4 = inner-half dentin. The full 0–4 scale was available, but because cavitated lesions were excluded at enrolment (see Specimens), no specimen received score 4; the analytical scale was therefore 0–3 by design, with no region excluded from analysis or merged into score 3, matching the ICDAS analytical range. This grading scheme has been used for caries-detection validation on extracted teeth [24] and ensured continuity with our previous DRS work [15].

### Spectral Preprocessing

Spectral data were processed in Python 3.11 (NumPy, SciPy, pandas), identically for tooth and material spectra. Calibrated reflectance R(λ) was computed, each spectrometer channel restricted to its high-SNR range (VIS 450–950 nm; NIR 950–1,700 nm), and the channels merged on a uniform 1-nm grid (1,251 pixels) with weighted averaging across the 950 nm overlap.

Each spectrum was quality checked: R(λ) had to fall within [−0.5, 10] for ≥95% of pixels. Sites whose ten spectra all failed QC were excluded; partial failures retained their valid spectra. Automated QC complemented the operator’s real-time inspection. In part 1, all 200 ROIs yielded complete data; in part 2, individual-spectrum failures were infrequent (1.7% of acquisitions) and three reference teeth measured in a single session (December 30, 2019) were excluded entirely for a session-specific calibration drift, with no other session showing systematic failures (online suppl. Table S2).

QC-passed spectra were smoothed with a Savitzky-Golay filter (second polynomial order, 11-point window) [25] and normalized by the standard normal variate (SNV) transformation [26] to compensate for multiplicative scattering arising from surface and probe-contact variability. The QC-passed spectra at each site were then averaged to yield one mean spectrum per site (the analytical unit). The pipeline is illustrated in online supplementary Figure S2.

### Statistical Analysis

Statistical analyses were performed in Python 3.11 (NumPy, SciPy, scikit-learn, statsmodels, pandas). Significance level α = 0.05; all confidence intervals are reported at 95%.

#### Statistical Analysis for Part 1 (Caries Detection)

Inter-examiner agreement was quantified by percentage agreement and Cohen’s kappa (unweighted, linear, quadratic) with 2,000-resample bootstrap 95% CIs, interpreted per Landis and Koch [27]. Method-vs.-histology agreement was assessed by exact match, quadratically weighted kappa with bootstrap 95% CI, and Spearman’s *ρ*.

Diagnostic accuracy was computed at two prespecified cutoffs – any caries (histology ≥D1) and dentin caries (histology = D3). Sensitivity, specificity, PPV, negative predictive value (NPV), and accuracy were reported with Wilson 95% CIs [28]. Because PPV and NPV depend on prevalence, both were recomputed across pretest prevalences of 5–70% with sensitivity and specificity held fixed (online suppl. Table S3). ROC analysis used the ordinal scores as continuous discriminators; AUC was computed empirically with DeLong 95% CI and pairwise AUC differences were tested with DeLong’s paired test [29]. For 200 ROIs at 60% prevalence, the 95% CI half-width is ≈ ±5 percentage points for sensitivities and specificities of 85–95%, adequate for a phase-II diagnostic study [30]. Because the two ROIs per tooth are not fully independent, a sensitivity analysis randomly selecting one ROI per tooth (1,000 repeats) was concordant with the primary analysis (online suppl. Table S1).

#### Statistical Analysis for Part 2 (Material Discrimination)

The site-mean spectrum (see Spectral Preprocessing) was the analytical unit; the dataset comprised 217 observations (180 material and 37 tooth-reference spectra). Classes were defined a priori by specimen identity (the four materials and sound enamel). Per-class mean spectra with 95% confidence bands were computed for calibrated and SNV-normalized representations; PCA on SNV spectra retained components for ≥95% cumulative variance.

Multi-class classification used partial least squares discriminant analysis (PLS-DA) [31] with one-hot encoding and argmax assignment; latent variables (2–15) were optimized by inner cross-validation. Outer 10-fold GroupKFold grouped all spectra from a cube or tooth in the same fold (73 groups: 4 × 9 cubes + 37 teeth), preventing train-test leakage. Performance was reported as overall accuracy (Wilson 95% CI), per-class sensitivity and specificity, confusion matrix, and macro-F1, and benchmarked against LDA on PCA scores and a default-parameter RBF support vector machine under the same grouped CV.

## Results

A total of 200 ROIs from 100 extracted teeth were assessed by all three modalities. Histological evaluation classified 80 ROIs (40.0%) as sound (histological score 0), 36 (18.0%) as D1, 52 (26.0%) as D2, and 32 (16.0%) as D3, with no ROI scored 4 (consistent with the inclusion criteria); the combined prevalence of any caries (D1–D3) was 60.0% (120/200). All 200 ROIs had complete data (online suppl. Table S4). The study flow is summarized in online supplementary Figure S1 (STARD flow diagram).

Inter-examiner ICDAS agreement was high: exact agreement 71.0% (142/200), unweighted κ = 0.607 (95% bootstrap CI: 0.521–0.688), linearly weighted κ = 0.751 (0.687–0.808), and quadratically weighted κ = 0.857 (0.806–0.897); the discrepancies between examiners were predominantly one-step rather than gross (Fig. 2b). The unweighted κ falls in the substantial range and the quadratically weighted κ in the almost-perfect range [27].

**Fig. 2.**
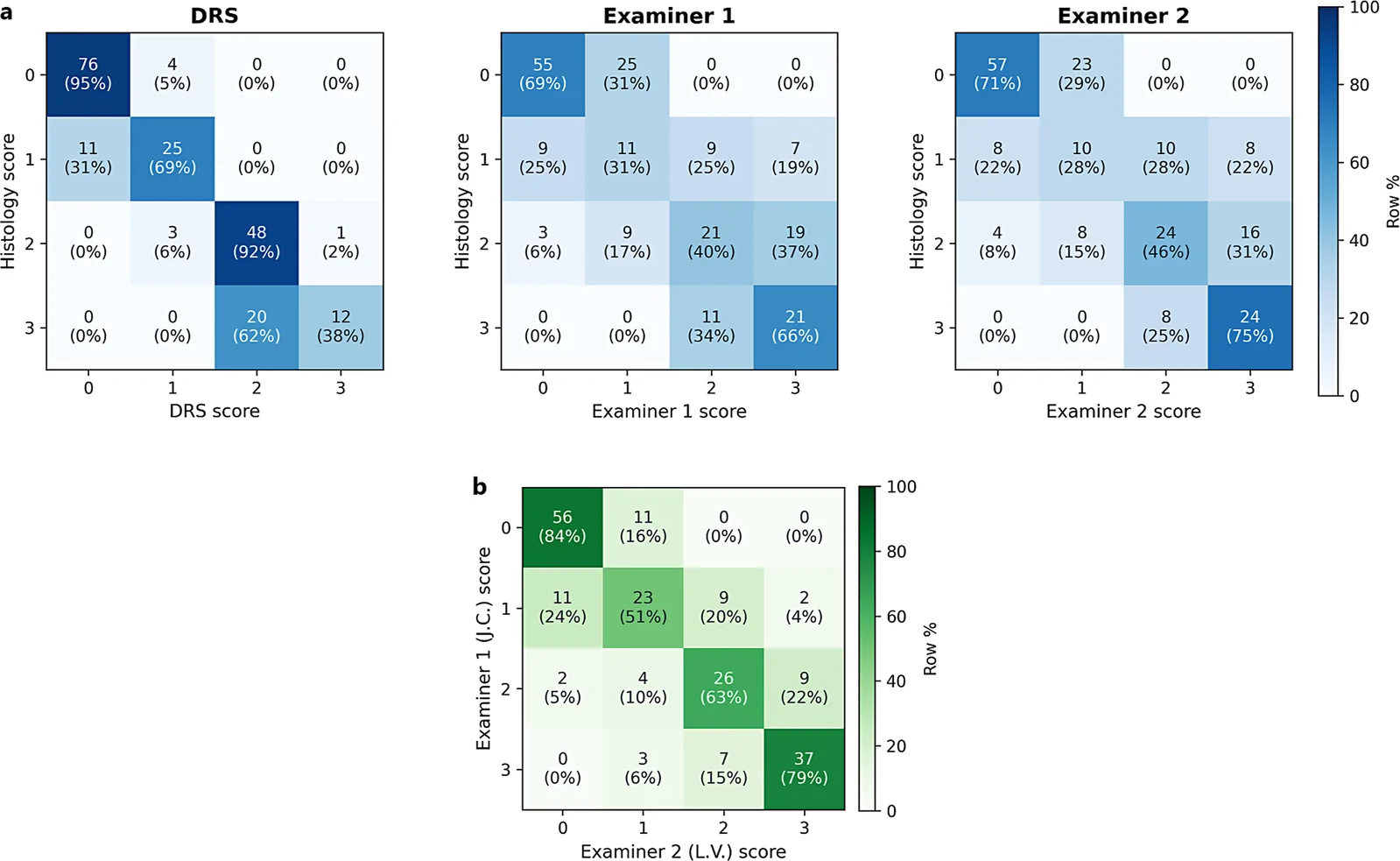
Confusion matrices against reference standards (part 1; *n* = 200 ROIs). **a** Each detection method (DRS, Examiner 1, Examiner 2) versus histology (histological scores 0–3). **b** Inter-examiner agreement (Examiner 1 vs. Examiner 2; exact agreement 71.0%, quadratically weighted κ = 0.857, 95% CI 0.806–0.897). Cells show absolute counts with within-row percentages; color intensity encodes row percentage. Diagonal cells indicate exact agreement. DRS, diffuse reflectance spectroscopy; Examiner 1 and 2, ICDAS examiners J.C. (1) and L.V. (2).

Method-versus-histology agreement was assessed by exact-match rate, quadratically weighted *κ*, and Spearman’s ρ (Table 2; Fig. 2a). DRS achieved 80.5% exact agreement (*κ* = 0.916, 95% CI: 0.890–0.939; *ρ* = 0.930, *p* < 0.001), whereas examiner 1 reached 54.0% (*κ* = 0.770, 0.713–0.819) and examiner 2 reached 57.5% (*κ* = 0.775, 0.714–0.829), DRS being the highest of the three.

**Table 2.** Agreement of each detection method with the histological reference standard (part 1)

Method	Exact match, %	Quadratically weighted κ (95% CI)	Spearman’s ρ
DRS	80.5	0.916 (0.890–0.939)	0.930
ICDAS examiner 1	54.0	0.770 (0.713–0.819)	0.831
ICDAS examiner 2	57.5	0.775 (0.714–0.829)	0.842

Confidence intervals for κ were computed by 2,000-resample bootstrap. All Spearman’s ρ values were significant at *p* < 0.001.

CI, confidence interval; DRS, diffuse reflectance spectroscopy; ICDAS examiner 1, J.C.; ICDAS examiner 2, L.V.; *κ*, Cohen’s kappa coefficient.

Diagnostic accuracy was computed at two cutoffs (Table 3; online suppl. Fig. S6). For any caries (histology ≥D1), DRS reached sensitivity 90.8% (95% CI: 84.3–94.8) and specificity 95.0% (87.8–98.0), against examiner 1 90.0% (83.3–94.2)/68.8% (57.9–77.8) and examiner 2 90.0% (83.3–94.2)/71.2% (60.5–80.0); DRS produced 4 false-positive classifications of sound surfaces, compared with 25 (examiner 1) and 23 (examiner 2). For dentin caries (D3 vs. not), the pattern reversed: DRS sensitivity (37.5%, 22.9–54.7) was lower than examiner 1 (65.6%) and examiner 2 (75.0%), while DRS specificity reached 99.4% (96.7–99.9), exceeding both examiners (84.5% and 85.7%). The confusion matrices (Fig. 2a) showed that DRS most often under-staged true D3 lesions as D2 (20 of 32 D3 ROIs) rather than misclassifying them as sound.

**Table 3.** Diagnostic accuracy of DRS and ICDAS at the any-caries and dentin thresholds (part 1)

Method	Sens. (95% CI)	Spec. (95% CI)	PPV (95% CI)	NPV (95% CI)	Acc. (95% CI)	TP/FP/TN/FN
*Any caries (histology ≥ D1 vs. sound)*						
DRS	90.8 (84.3–94.8)	95.0 (87.8–98.0)	96.5 (91.6–98.5)	87.4 (78.7–92.9)	92.5 (88.0–95.4)	109/4/76/11
ICDAS examiner 1	90.0 (83.3–94.2)	68.8 (57.9–77.8)	81.2 (73.8–86.8)	82.1 (70.8–89.7)	81.5 (75.6–86.2)	108/25/55/12
ICDAS examiner 2	90.0 (83.3–94.2)	71.2 (60.5–80.0)	82.4 (75.2–87.9)	82.6 (71.7–90.0)	82.5 (76.7–87.1)	108/23/57/12
*Dentin caries (histology = D3 vs. not)*						
DRS	37.5 (22.9–54.7)	99.4 (96.7–99.9)	92.3 (66.5–98.7)	89.3 (84.2–92.9)	89.5 (84.6–93.0)	12/1/167/20
ICDAS examiner 1	65.6 (48.3–79.6)	84.5 (78.3–89.2)	44.7 (31.4–58.8)	92.8 (87.6–95.9)	81.5 (75.6–86.2)	21/26/142/11
ICDAS examiner 2	75.0 (57.9–86.7)	85.7 (79.6–90.2)	50.0 (35.8–64.2)	94.7 (90.0–97.3)	84.0 (78.3–88.4)	24/24/144/8

Values are percentages (%) with 95% Wilson score confidence intervals in parentheses.

ICDAS examiner 1, J.C.; ICDAS examiner 2, L.V.; Acc., accuracy; CI, confidence interval; DRS, diffuse reflectance spectroscopy; FN, false negative; FP, false positive; NPV, negative predictive value; PPV, positive predictive value; Sens., sensitivity; Spec., specificity; TN, true negative; TP, true positive.

ROC analysis (Fig. 3) yielded AUC values of 0.946 (95% CI: 0.918–0.974) for DRS, 0.908 (0.870–0.946) for examiner 1, and 0.914 (0.877–0.951) for examiner 2 at the any-caries cutoff; DeLong’s test showed DRS to have a significantly higher AUC than examiner 1 (ΔAUC = +0.038, *p* = 0.027), while the difference versus examiner 2 was not significant (ΔAUC = +0.032, *p* = 0.076); a clustering sensitivity analysis (one ROI per tooth, 1,000 iterations) gave concordant results (online suppl. Table S1). At the dentin-caries cutoff, AUCs were 0.906, 0.865, and 0.885, respectively; pairwise differences were not statistically significant (*α* = 0.05).

**Fig. 3.**
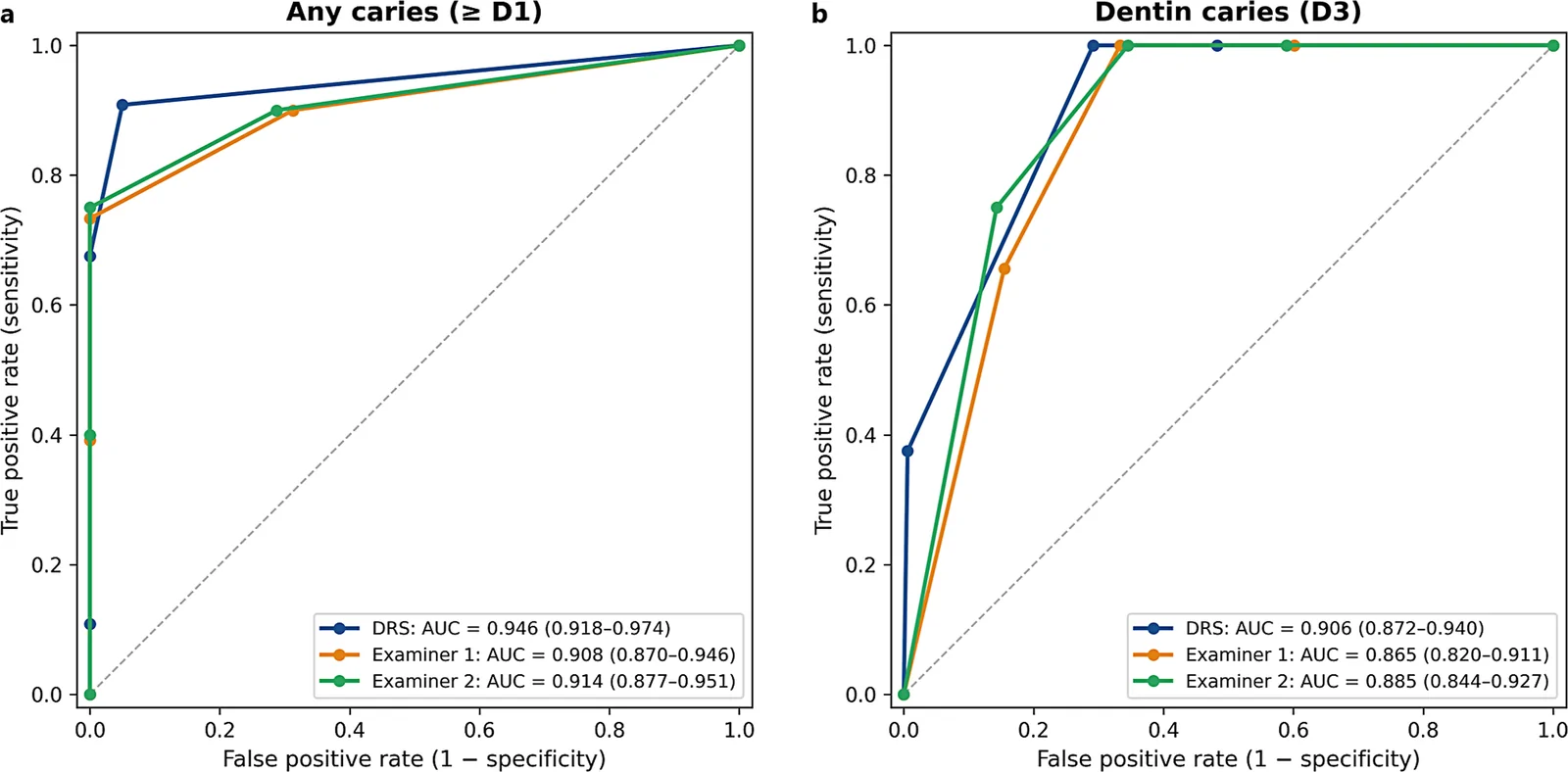
Empirical receiver operating characteristic (ROC) curves for DRS, Examiner 1, and Examiner 2 against the histological reference (part 1; *n* = 200 ROIs). **a** Any caries (histology ≥D1) versus sound. **b** Dentin caries (histology = D3) versus not. Ordinal scores were treated as continuous discriminators. AUC is shown with 95% DeLong confidence intervals; the dashed diagonal indicates chance. DRS achieved a significantly higher AUC than Examiner 1 at the any-caries cutoff (DeLong *p* = 0.027); other comparisons were not significant. AUC, area under the curve; DRS, diffuse reflectance spectroscopy; Examiner 1 and 2, ICDAS examiners J.C. and L.V.; ROI, region of interest.

The preprocessed part 2 dataset comprised 217 site-mean spectra: 180 material sites and 37 tooth-reference sites. Two categories of QC exclusion applied (online suppl. Table S2). First, 37 isolated spectra (1.7% of 2,200 acquisitions) failed individually and were dropped from per-site averaging. Second, all ten spectra of three tooth-reference specimens failed because of a session-specific calibration drift (December 30, 2019; see Specimens), excluding those specimens (30 spectra). Total QC exclusions were therefore 67 of 2,200 acquisitions (3.0%), leaving 73 specimen groups (4 × 9 cubes + 37 teeth) for cross-validation.

The four material classes exhibited distinct spectral fingerprints across 450–1,700 nm (Fig. 4a). Riva LC showed the highest mean reflectance, peaking near 700–800 nm; G-aenial Anterior and Enamel Plus HRi exhibited the lowest mean reflectance and similar overall shapes (mean Pearson r between-class means = 0.97); RelyX Unicem showed an intermediate, monotonically decreasing profile. Tooth-reference spectra had a markedly wider 95% confidence band than any material class. Principal component analysis on SNV spectra required three components for ≥95% cumulative variance (97.9%; online suppl. Fig. S3). The four material classes formed compact, well-separated clusters; tooth-reference observations were distributed more widely along PC2 and overlapped predominantly with the Enamel Plus HRi cluster.

**Fig. 4.**
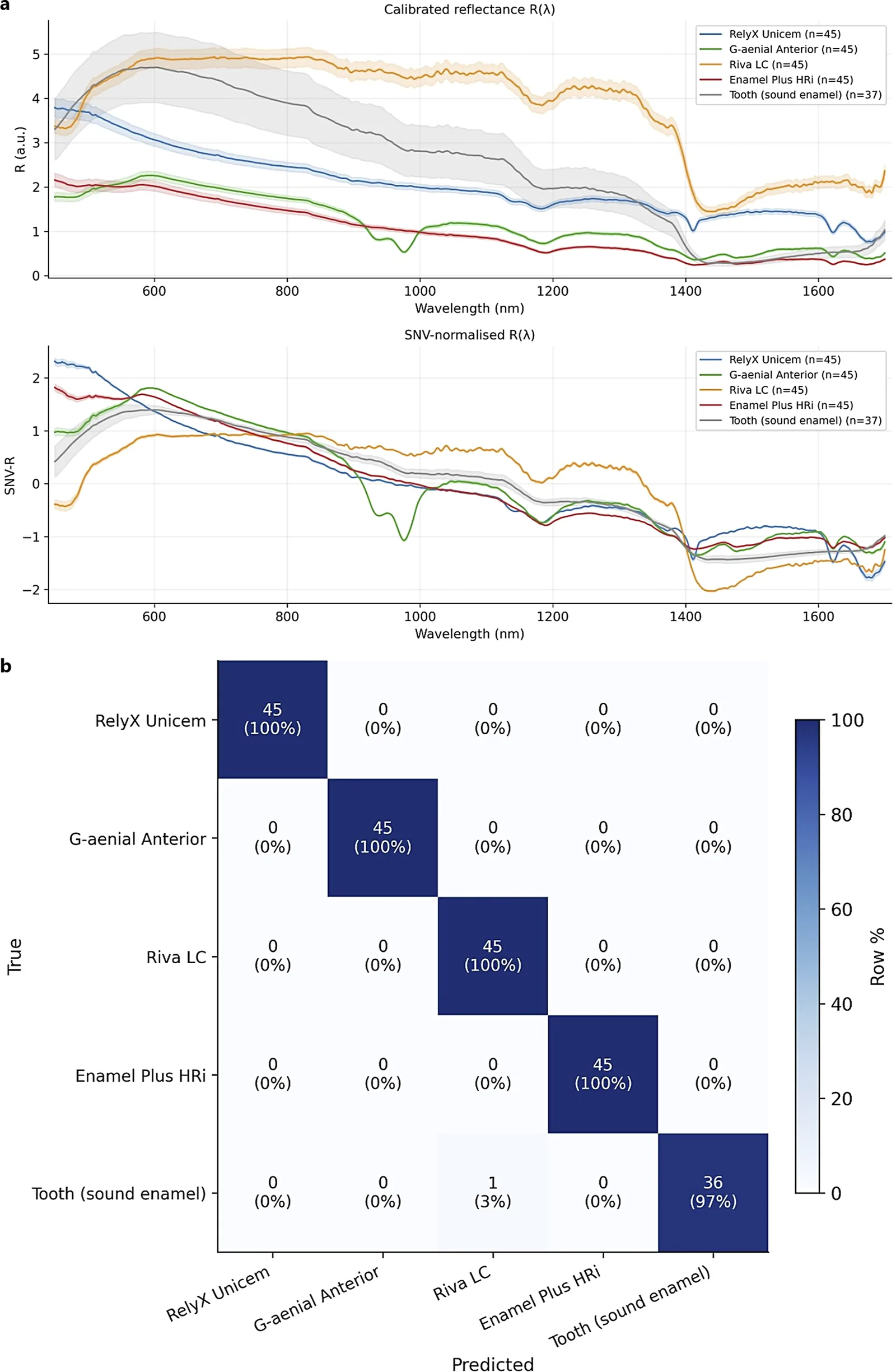
Spectral discrimination of restorative materials (part 2; *n* = 217 sites). **a** Mean calibrated reflectance R(λ) (top) and corresponding SNV-normalized spectra (bottom), each with 95% confidence bands, for the four materials and sound enamel (RelyX Unicem, blue; G-aenial Anterior, green; Riva LC, orange; Enamel Plus HRi, red; sound enamel, gray); working range 450–1700 nm. **b** PLS-DA confusion matrix from specimen-grouped 10-fold cross-validation (73 groups): rows, a-priori true class; columns, predicted class; cells show counts with within-row percentages. Overall accuracy 99.5% (95% Wilson CI: 97.4–99.9), macro-F1 0.995; one sound-enamel site was misclassified as Riva LC. PLS-DA, partial least squares discriminant analysis.

PLS-DA classification under specimen-grouped 10-fold cross-validation achieved overall accuracy 99.5% (95% CI: 97.4–99.9) and macro-F1 0.995 (Table 4; online suppl. Fig. S4). All four material classes achieved sensitivity and specificity of 100%, except Riva LC (specificity 99.4% owing to a single tooth-reference observation classified as Riva LC). The tooth-reference class reached sensitivity 97.3% (36/37) and specificity 100%; the single misclassified tooth appeared as an outlier in the PCA scatter (online suppl. Fig. S3), closer to the Riva LC cluster than to the main tooth-reference distribution. The full 5 × 5 confusion matrix is shown in Figure 4b. The number of latent variables selected by inner CV ranged from 5 to 10 across the 10 outer folds (median 8).

**Table 4.** Per-class diagnostic metrics of PLS-DA classification under specimen-grouped 10-fold cross-validation (part 2; *n* = 217 sites)

Class	TP/FN/FP/TN	Sensitivity, % (95% CI)	Specificity, % (95% CI)	F1
RelyX Unicem	45/0/0/172	100.0 (92.1–100.0)	100.0 (97.8–100.0)	1.000
G-aenial Anterior	45/0/0/172	100.0 (92.1–100.0)	100.0 (97.8–100.0)	1.000
Riva LC	45/0/1/171	100.0 (92.1–100.0)	99.4 (96.8–99.9)	0.989
Enamel Plus HRi	45/0/0/172	100.0 (92.1–100.0)	100.0 (97.8–100.0)	1.000
Tooth (sound enamel)	36/1/0/180	97.3 (86.2–99.5)	100.0 (97.9–100.0)	0.986

Overall classification accuracy: 99.5% (95% Wilson CI 97.4–99.9%); macro-averaged F1 = 0.995. Sensitivity and specificity are point estimates with 95% Wilson score confidence intervals in parentheses.

CI, confidence interval; F1, F1 score; FN, false negative; FP, false positive; PLS-DA, partial least squares discriminant analysis; TN, true negative; TP, true positive.

Under the same grouped cross-validation, LDA on PCA scores (≥95% variance) reached accuracy 92.6% and macro-F1 0.914, and a default-parameter RBF support vector machine reached 88.0% and 0.843, respectively (online suppl. Fig. S5). The consistent ordering – PLS-DA > LDA-PCA > RBF-SVM – was preserved across all per-class metrics (online suppl. Fig. S5).

## Discussion

DRS matched or exceeded trained ICDAS examiners across two complementary diagnostic tasks: agreement with a histological reference for occlusal caries detection, and discrimination of four freshly prepared restorative materials from sound enamel. Its principal advantage over visual scoring was a substantial reduction in false-positive classifications of sound surfaces, while material classes were separated with high accuracy under leakage-controlled cross-validation. These proof-of-principle findings position DRS as a candidate optical adjunct, for occlusal caries detection and for differentiating restorative materials from sound enamel, that now requires in vivo evaluation.

The most clinically meaningful finding of part 1 is the markedly higher specificity of DRS at the any-caries threshold than either ICDAS examiner. Visual examination over-diagnoses enamel demineralization in the presence of post-eruptive maturation or stains [6, 32]; because DRS quantifies bulk optical properties of the mineralized matrix rather than surface appearance, such confounders contribute only marginally to the spectral features used for classification, providing a mechanistic basis for the specificity gain over fluorescence-based adjuncts such as DIAGNOdent [33, 34]. The DRS specificity falls at the high end of the pooled visual-inspection specificities reported by Gimenez et al. [5] (0.57–0.99) and exceeds the pooled OCT specificity in the Cochrane review of Macey et al. [7] (0.83), while requiring far simpler instrumentation. The DRS sensitivity is comparable to pooled OCT sensitivity and well above NIR transillumination in the same review [7].

Predictive values depend on prevalence, so the values reported here reflect the 60% any-caries prevalence of this enriched in vitro sample; caries prevalence varies widely across populations and is generally lower in routine screening [1]. Recomputing PPV and NPV across a 5–70% prevalence range at fixed sensitivity and specificity (online suppl. Table S3) shows the expected pattern: NPV rises and PPV falls as prevalence decreases. Because DRS combines high specificity with high sensitivity, its PPV stays clinically useful even at low prevalence – about 82% at a prevalence of 20%, against roughly 42–44% for the visual examiners at the same prevalence – while NPV remains above 97%. This reinforces the rationale that for a caries adjunct, high specificity and PPV matter as much as sensitivity.

DRS most often classified true dentin lesions (D3) as D2 (20 of 32 under-staged by one category) rather than missing them; DRS sensitivity at the dentin cutoff was consequently lower than that of both examiners. We interpret this as a depth-of-information limit rather than a diagnostic shortcoming: the VIS-NIR probing volume of the current probe is shallower than the histological extent of an outer-half dentin lesion [35, 36]. Clinically, however, under-staging deeper lesions is not without consequence – a DRS-only reading could under-estimate lesion depth and, if acted on in isolation, delay operative care that a dentin lesion may warrant. Reassuringly, no D3 lesion was classified as sound, so within an adjunctive role – flagging demineralization for further, depth-sensitive assessment rather than replacing it – an under-staged but flagged lesion still prompts evaluation, and DRS should complement, not replace, methods sensitive to lesion depth.

Inter-examiner ICDAS agreement reached almost-perfect levels (quadratically weighted κ = 0.857) despite no hands-on pre-study calibration, with both examiners trained only via the standardized ICDAS Foundation e-learning module [20, 32]. This supports structured online training as a pragmatic alternative to in-person calibration when post hoc agreement statistics are reported.

The high PLS-DA accuracy was achieved under a stringent specimen-grouped cross-validation scheme – all spectra from a given cube or tooth assigned to the same fold – preventing the information leakage that can inflate accuracy in spectral classification [37] and was independently reproduced with a separately implemented pipeline yielding a bit-identical confusion matrix. This performance should be read as proof-of-principle: it was obtained on freshly prepared, single-shade specimens under controlled optics and is unlikely to transfer unchanged to the aged, stained, or multi-shade restorations encountered clinically. Riva LC, a resin-modified glass-ionomer with fluoroaluminosilicate glass filler, showed the highest mean reflectance across the VIS and short-wave NIR range, dominated by particle scattering. The two resin-based composites exhibited the lowest reflectance and similar spectral shapes (mean Pearson *r* = 0.97), reflecting their shared methacrylate matrix. The overlap between sound enamel and Enamel Plus HRi in PCA space is consistent with the biomimetic optical design of this enamel-shade composite, intended to match the refractive index and scattering profile of natural enamel.

Because part 2 used freshly polymerized, planar block specimens, the findings demonstrate spectral separability under controlled conditions rather than the ability to verify restoration identity, placement, or setting in the clinic. Generalization to aged restorations, which undergo surface staining, biofilm acquisition, and filler hydrolysis, requires separate validation, as noted below.

Strengths of the study include the use of histology as the reference standard for caries detection rather than radiographic validation, the explicit specimen-grouped cross-validation scheme protecting part 2 from optimistic bias, and full reporting in accordance with the STARD 2015 framework [18, 19]. Additional strengths are the integration of two diagnostic arms within a single methodological framework and the independent reproduction of the PLS-DA result with a separately implemented analysis pipeline.

Several limitations apply. First, extracted teeth and freshly polymerized specimens cannot reproduce the intraoral environment: in vivo, a salivary and pellicle film, gingival fluid, blood, and plaque add absorbing and scattering layers over the surface, ambient and operatory light raises the optical background, and patient and probe movement introduces variability in fiber-to-surface distance and angle. Each factor alters the acquired reflectance and may shift or blur the spectral features on which classification depends, so the present accuracy should be regarded as an upper bound that in vivo studies must confirm. Second, the four materials represent a small, single-shade, unaged subset; discrimination of same-class or aged restorations remains untested. Third, histology was scored by a single experienced histologist, with intra-rater reliability documented previously rather than re-assessed [15]. Fourth, the number of D3 lesions (*n* = 32) limits the precision of the dentin-caries sensitivity estimate. Fifth, three of 40 tooth-reference specimens were excluded by QC, all from a single session likely affected by calibration drift, mitigated by routine reference-tooth verification. Sixth, all measurements were single-operator, single-laboratory; multicenter reproducibility remains to be established. Seventh, only posterior occlusal surfaces were assessed; smooth, approximal, and buccal-cervical morphologies warrant separate evaluation. Storage is unlikely to have biased these findings: enamel retains largely stable optical properties in 0.5% chloramine-T, with only minor changes emerging after several months [38], and the reflectance signal used by DRS is less storage-sensitive than the fluorescence readings of some optical caries devices [39].

Several priorities follow for clinical translation. First, in vivo validation in adult and pediatric populations is essential, because saliva, pellicle, patient movement, and intraoral optical variability will attenuate the controlled performance reported here. Second, because DRS under-stages deeper lesions, it should be paired with a modality better suited to lesion depth, such as NIR transillumination or radiography, which face the complementary problem of resolving dentin from enamel [40]; hybrid protocols could still combine the sensitivity of visual scoring for advanced lesions with the specificity of DRS for early ones. Third, lesion activity deserves attention, as active and arrested lesions differ in porosity and light scattering, and optical activity assessment is an emerging capability for in vivo DRS work [41]. Fourth, the spectral library should be expanded to further material classes, brands, shades, and specimens of varying clinical age, since aging and staining measurably alter restorative-material optics [42]. Finally, embedding DRS in a point-of-care workflow with real-time processing will show whether these gains support clinical decisions.

Within the limitations of an in vitro design, DRS with appropriate preprocessing and supervised classification discriminated early occlusal caries and four freshly prepared restorative materials from sound enamel, with markedly higher specificity than visual ICDAS scoring. These are proof-of-principle results: their principal potential benefit – fewer unnecessary operative interventions – remains to be demonstrated in vivo, where saliva, plaque, and intraoral variability will test the method’s robustness. DRS is therefore best positioned as an adjunct to, not a replacement for, established caries assessment.

## Acknowledgments

The authors thank the Institute of Dental Medicine and the Institute of Histology and Embryology, First Faculty of Medicine, Charles University, for assistance with specimen processing. The authors would like to acknowledge Philips Research for providing the prototype of the DRS device. During the preparation of this manuscript, the authors used AI-assisted tools for language editing and for support with data analysis and figure preparation. The authors reviewed and verified all content and take full responsibility for the integrity of the work.

## Statement of Ethics

This study protocol was reviewed and approved by the Ethics Committee of the General University Hospital, Approval No. 613/18 S-IV (2018) and 1/23 GIP (2023). All procedures involving human biological material (extracted teeth) were performed in accordance with the Declaration of Helsinki (1964, as revised in 2013). Extracted teeth used in this in vitro study were obtained from patients undergoing routine clinical extractions; written informed consent for the use of extracted teeth in dental research was obtained from all donors prior to extraction as part of the standard institutional consent procedure. All teeth were anonymized at the point of collection and could not be traced back to individual donors.

## Conflict of Interest Statement

The authors have no conflicts of interest to declare. The CONSOLE10 dual-spectrometer device used in this study was provided by Philips Research for academic research purposes only; Philips Research had no role in study design, data acquisition, data analysis, interpretation, or the decision to submit this manuscript for publication.

## Funding Sources

This study was supported by institutional research funding of the First Faculty of Medicine, Charles University in Prague. This work was supported by the Cooperatio Program, research area “Dental Medicine.” The funders had no role in the design of the study, data collection, data analysis, interpretation, manuscript writing, or the decision to submit the manuscript for publication.

## Author Contributions

J.C.: conceptualization, methodology, investigation (DRS acquisition, ICDAS scoring as examiner 1), data curation, formal analysis, visualization, writing – original draft, review, and editing, and project administration. L.V.: investigation (ICDAS scoring as examiner 2), validation, and writing – review and editing. L.H.: conceptualization, supervision, resources, and writing – review and editing. T.K.: investigation (histological processing and scoring), methodology (histology protocol), resources, and writing – review and editing. A.P.: methodology (spectral preprocessing and machine learning pipeline), software, formal analysis, validation, and writing – review and editing. All authors have read and approved the final manuscript.

## Data Availability

Representative preprocessed spectral data acquired with the CONSOLE10 prototype instrument are available as a text file from the corresponding author (J.C.) upon reasonable request, together with the de-identified histological and ICDAS scores, the spectral preprocessing pipeline, and the full statistical and classification analysis code, sufficient to demonstrate and verify the analytical workflow. Raw instrument acquisitions are stored in a proprietary binary format requiring extensive post-processing and are not shared. Full datasets are not publicly shared, in compliance with institutional data-protection policies and the conditions of the ethics approval.
